# CC chemokines are differentially expressed in Breast Cancer and are associated with disparity in overall survival

**DOI:** 10.1038/s41598-019-40514-9

**Published:** 2019-03-08

**Authors:** Jeronay K. Thomas, Hina Mir, Neeraj Kapur, Sejong Bae, Shailesh Singh

**Affiliations:** 1 Department of Microbiology, Biochemistry and Immunology; Cancer Health Equity Institute, Morehouse School of Medicine, Atlanta, GA 30310 USA; 2 Division of Preventive Medicine, Department of Medicine, University of Alabama at Birmingham, Birmingham, AL 35294 USA

## Abstract

Despite recent advances, breast cancer (BrCa) still affects many women and the impact is disproportional in African Americans (AA) compared to European Americans (EA). Addressing socioeconomic and behavioral status has not been enough to reduce disparity, suggesting contribution of biological differences in BrCa disparity. Our laboratory was first to show involvement of CC chemokines in BrCa. In this study, using ONCOMINE, TCGA, bc-GenExMiner and KMplotter, we examined the association of CC chemokines in BrCa outcomes and disparity. We show over-expression of CCL5, -7, -11, -17, -20, -22 and -25 in BrCa tissues. High mRNA levels of CCL7, -8, -17, -20 and -25 predicted a decrease in overall survival (OS). CCL7 and CCL8 were associated with decreased relapse-free survival. Expression of CCL17 and CCL25 was associated with decreased OS in AA. In EA, CCL8 was associated with decreased OS. Expression of CCL5, -7, -8, -17, -20 and -25 was highest in TNBC. Expression of CCL11 and CCL22 was associated with HER2. CCL7, -8, -17, -20 and -25 were elevated in AAs. In conclusion, our analysis suggests significant association of CC-chemokines in BrCa progression, OS and disparate disease outcome in AA compared to EA patients.

## Introduction

Breast cancer (BrCa) alone accounts for 30% of all new cancers diagnosed in women^1^ and is the second leading cause of cancer related deaths in women after lung cancer^1^. Complete etiology of BrCa is yet to be defined, however lifestyle, genetic and environmental factors are often associated with this multifactorial disease^2^. Undefined etiology and heterogeneity of BrCa are major challenges in developing definitive therapeutics. Among all BrCa types Triple Negative Breast Cancer (TNBC), which lacks estrogen receptor (ER), progesterone receptor (PR) and human epidermal growth factor receptor 2 (HER2) is the most lethal type. Moreover, this subtype is further elucidated into 6 subtypes identified by cluster analysis^3^. Despite higher incidence of BrCa in European Americans (EA), African Americans (AA) are more often diagnosed with TNBC and have a worst prognosis compared to EA with TNBC^4,5^. Studies have shown that BrCa progression and development is highly influenced by inflammation^6^ and the immune system^7^. It is crucial to identify biomarkers, which play a role in these processes, in order to develop novel personalized treatments for BrCa patients.

Chemokines are a large family of small cytokines, which are classified into 4 different subgroups (C, CC, CXC and CX3C), based on cysteine residues. These molecules are responsible for immune cell trafficking and shaping the immune system. They also play a role in inflammation. It is now well established that chemokines and chemokine receptors are expressed by cancer cells and play a significant role in cancer progression and therapeutic outcomes. Our laboratory and others have shown association of chemokines in various cancers^8–15^. Among all known chemokines and chemokine receptors, CXC chemokines are well studied in cancer^8,9,11–16^. However our laboratory was first to show association of CC chemokine receptor-9 (CCR9) and its natural ligand CCL25 in various cancers, including BrCa^10,17–21^.

Higher incidence of BrCa among AA at a younger age and higher mortality of AA with TNBC compared to EA indicate contribution of race specific biological differences to the disparity in disease and therapeutic outcome of the disease. Therefore, it is imperative to define these racial differences in BrCa molecular footprints to address the observed disparity. There are only a few studies available addressing this issue related to chemokines. Recent analysis, have shown association of CXC chemokines with BrCa^22^. In this study we have utilized large-scale bioinformatics to ascertain CC chemokine expression in BrCa patients based on clinical parameters, respective prognosis and have presented evidence suggesting association of CC chemokines with BrCa progression, TNBC and racial disparity in overall survival (OS).

## Results

### CC Chemokines are elevated in BrCa tissues

Using the ONCOMINE database (TCGA breast invasive carcinoma dataset), out of all known CC chemokines 7 CC chemokines’ [(CCL5 (FC = 1.6, p = 8.6e^−05^), CCL7 (FC = 4.5, p = 1.43e^−14^), CCL11 (FC = 5.5, p = 1.29e^−31^), CCL17 (FC = 2.0, p = 3.32e^−09^), CCL20 (FC = 2.2, p = 2.07e^−06^), CCL22 (FC = 1.44, p = 1.5e^−04^) and CCL25 (FC = 1.6, p = 1.33e^−08^)] mRNA were significantly (fold change ≥1.4 and p-value ≤ 0.04) elevated in BrCa compared to normal tissues (Table 1). Furthermore, we elucidated association of these chemokines with histological subtype of BrCa (Table 2). We used several datasets within ONCOMINE to establish the association of CC chemokines with histological subtypes. Curtis and Ginestier^23,24^ datasets showed that CCL5 was expressed in medullary BrCa with a fold change ranging from 3–8 between Curtis and Ginestier data sets, when compared to other BrCa histological types (p = 0.004 and p = 1.11e^−07^). The Bittner breast dataset showed CCL5 elevated in ductal (fold change of 1.4, p = 0.02) carcinoma. The TCGA dataset showed CCL7 to be 2 fold greater in ductal BrCa when compared to other BrCa types (p = 2.52e^−06^). The Radvanyi^25^ dataset reported CCL11 to have a 3 fold change in ductal carcinoma, compared to other types (p = 1.09e^−04^). CCL17 was 1.3 fold greater in lobular BrCa (p = 0.03) in the TCGA dataset. Lu dataset displayed 3.5 fold higher CCL20 (p = 9.88e^−05^) in ductal carcinoma^26^, whereas in Ginestier dataset 6 fold change in CCL20 was observed (p = 0.012) in medullary BrCa^24^. There was only 1 fold change in CCL22 expression in ductal carcinoma observed in Curtis dataset (p = 2.34e^−09^)^23^, while Radvanyi conveyed a 1.9 greater fold change (p = 0.035) in lobular BrCa for CCL22^25^. Perou dataset indicated 1.7-fold change in CCL25 (p = 0.034) in Lobular BrCa^27^ and as per TCGA this change was 1.4 fold (p = 0.015) in ductal carcinoma *in situ*.

**Table 1:** Significant changes of CC- Chemokines among BrCa tissues in comparison to normal tissues using ONCOMINE.

	Fold Change	p-value	t-test
CCL1	1.2	0.011	2.332
CCL2	−1.195	0.932	−1.498
CCL3	1.181	0.164	0.984
CCL4	1.024	0.414	0.218
**CCL5**	1.598	8.60E-05	3.865
**CCL7**	4.522	1.43E-14	8.62
CCL8	−1.061	0.651	−0.39
**CCL11**	5.535	1.29E-31	15.426
CCL13	−1.393	0.986	−2.226
CCL14	−1.12	0.989	−2.325
CCL15	−1.12	0.989	−2.325
CCL16	−1.161	0.996	−2.655
**CCL17**	1.957	3.32E-09	6.201
CCL18	1.236	0.106	1.255
CCL19	−1.147	0.871	−1.138
**CCL20**	2.16	2.07E-06	4.804
CCL21	−1.957	1	−6.17
**CCL22**	1.438	1.47E-04	3.72
CCL23	−1.429	0.999	−3.154
CCL24			
**CCL25**	1.598	1.33E-08	5.909
CCL26	−1.209	0.989	−2.329
CCL27	−1.204	1	−3.648
CCL28	−6.33	1	−9.015

Fold Change denotes the folds difference in CC chemokine mRNA expression in BrCa in comparison to normal tissues. The p value represents the statistical significance of the difference in the mean of the two groups as analyzed using t-test. Bold represents significant RNA expression with a cut off of: fold change ≥1.4 and p-value ≤ 0.04 using t-test.

**Table 2:** Significant changes of CC chemokines among different histological types of BrCa.

Data set	Tissue Type (No. of Cases)	CCL5		CCL7		CCL11		CCL17		CCL20		CCL22		CCL25	
		FC	p	FC	p	FC	p	FC	p	FC	p	FC	p	FC	p
Bittner	Ductal (n = 260)	**1.4**	**0.02**	**1.5**	**0.002**	**1.4**	**0.008**	—	—	**1.5**	**0.008**	1.1	0.3	−1.1	0.7
	Lobular (n = 38)	−1.1	0.7	−1.6	1	1.3	0.25	—	—	−1.6	1	−1.2	1	−1.1	0.8
Boersma^78^	Ductal (n = 83)	1.1	0.4	1.2	0.014	1.3	0.003	1	0.3	1.1	0.3	−1.2	0.7	1.3	0.008
	Lobular (n = 10)	1.1	0.4	−1.2	1	−1.3	1	−1	0.7	−1.1	0.7	1.2	0.2	−1.3	1
Curtis^23^	Ductal (n = 1556)	1	0.36	1	0.002	1	0.02	—	—	1	1.3e^−4^	1.1	2.3e^−9^	1	0.4
	Medullary (n = 32)	**3.3**	**1e** ^**−7**^	1.3	0.007	1	0.2	—	—	**1.4**	**0.002**	1.2	0.02	1.2	4.1e^−4^
	Lobular (n = 148)	1.1	0.14	−1.1	1	−1	1	—	—	−1.1	1	−1.1	1	−1	1
Desmedt^79^	Ductal (n = 158)	1.5	0.06	1.3	0.09	1.1	0.3	—	—	1.2	0.2	1.1	0.3	1	0.4
	Lobular (n = 13)	−1.2	0.9	−1.4	1	−1.1	0.8	—	—	−1.2	0.7	−1.3	0.9	−1	0.6
Esserman^80^	Ductal (n = 105)	1.1	0.4	**1.9**	**0.04**	1.3	0.05	1.1	0.4	−1.1	0.7	−1.1	0.7	1	0.4
	Lobular (n = 8)	1.1	0.4	−2.3	1	−1.2	0.9	−1.2	00.8	1.2	0.3	1.1	0.3	1	0.4
Ginester^24^	Ductal (n = 45)	−3.8	1	−1.3	0.8	−1.3	0.74	1	0.23	−3.2	1	1.2	0.84	−1.1	0.7
	Medullary (n = 5)	**8.5**	**0.004**	1.8	0.07	1.8	0.13	1	0.335	**6**	**0.012**	1.2	0.11	1.2	0.08
Lu^26^	Ductal (n = 95)	1.2	0.13	1.4	0.04	1.1	0.2	—	—	**3.5**	**9.9e** ^**−5**^	1	0.5	−1	0.5
	Lobular (n = 19)	−1.2	0.8	−1.3	0.9	−1.1	0.8	—	—	−2.7	1	1.3	0.2	−1	0.5
MA2^81^	Ductal (n = 32)	−1.6	0.9	1	0.3	−1.1	0.8	1.1	0.14	−1.1	0.6	1.1	0.4	−1.1	0.7
	Lobular (n = 3)	3.7	0.06	−1	0.8	−1.1	0.7	−1	0.7	−1.1	0.8	−1.1	0.6	−1	0.6
MA3^81^	Ductal (n = 51)	−1.2	0.7	1	0.4	−1.2	0.8	1.1	0.3	−1.1	0.6	−1.2	0.9	1	0.4
	Lobular (n = 3)	1.8	0.2	1	0.3	1.4	0.04	1.3	0.2	−1.1	0.6	1.9	0.1	1.1	0.4
MA4^82^	Ductal (n = 18)	−1	0.61	—	—	−1.1	0.8	−1.1	1	−1.2	0.6	−1.2	1	−1	0.5
	Ductal *in situ* (n = 20)	1.9	0.04	—	—	1.1	0.2	1.1	0.04	1.2	0.4	1.2	0.07	1	0.5
Perou^27^	Ductal (n = 54)	1.1	0.4	−1	0.5	1	0.4	—	—	—	—	—	—	−1.7	1
	Lobular (n = 4)	−1	0.7	1	0.5	−1	0.6	—	—	—	—	—	—	**1.7**	**0.03**
Pollack^83^	Ductal (n = 105)	−1.1	0.63	1.6	0.1	1.3	0.3	—	—	—	—	—	—	−2.5	0.8
	Lobular (n = 8)	1.1	0.4	−1.6	0.9	−1.3	0.8	—	—	—	—	—	—	2.5	0.2
Radvanyi^25^	Ductal (n = 26)	—	—	—	—	**2.8**	**1**e^−4^	1.3	0.3	1.7	0.4	1.3	0.2	−1.3	0.7
	Lobular (n = 4)	—	—	—	—	−1.7	0.9	1.5	0.2	−1.1	0.8	**1.9**	**0.04**	1.1	0.3
	Ductal *in situ* (n = 2)	—	—	—	—	−2.8	1	−1.3	0.7	−1.7	0.6	1.8	0.1	1.3	0.3
Schuetz^84^	Ductal (n = 5)	1.3	0.25	—	—	1.2	0.03	−1.1	0.9	−1.4	1	−1.1	0.9	1	0.4
	Ductal *in situ* (n = 5)	−1.3	0.73	—	—	−1.2	1	1.1	0.15	**1.4**	**0.02**	1.1	0.15	−1	0.6
Sorlie^85^	Ductal (n = 66)	1.5	0.1	1.2	0.2	1.5	0.08	—	—	—	—	—	—	−2	0.9
	Lobular (n = 5)	−1.2	0.6	−1.2	0.8	−1.5	0.83	—	—	—	—	—	—	2.8	0.2
Sorlie2^86^	Ductal (n = 132)	1.2	0.3	—	—	1.4	0.05	—	—	—	—	—	—	—	—
	Lobular (n = 11)	−1	0.5	—	—	−1.5	0.92	—	—	—	—	—	—	—	—
Tabchy^87^	Ductal (n = 163)	−1.3	0.9	1.1	0.12	−1	0.8	−1	0.81	1.2	0.2	−1.1	0.9	−1.1	0.9
	Lobular (n = 7)	1.5	0.11	−1	0.8	1.1	0.09	1.1	0.3	−1.2	0.7	1.1	0.1	1.2	0.1
TCGA	Ductal (n = 392)	1.7	0.2	**2.1**	**2.5**e^−6^	−2.4	0.8	1.5	0.2	−1.4	0.7	−1.1	0.6	1.2	0.2
	Lobular (n = 36)	1.4	0.04	−1.8	1	−1	0.7	1.3	0.03	−1.1	0.8	1	0.4	−1	0.5
	Ductal *in situ* (n = 3)	—	—	—	—	2.1	0.1	−1.3	0.7	2	0.2	2.6	0.1	**1.4**	**0.02**
Turashvili^88^	Ductal (n = 5)	1.5	0.3	−1.6	0.8	2	0.2	−1	0.5	−1.1	0.6	1.1	0.4	−1	0.5
	Lobular (n = 5)	−1.1	0.5	1.6	0.2	−2	0.8	1	0.5	1.1	0.4	−1.1	0.5	1	0.5
Zhao^89^	Ductal (n = 39)	1.3	0.01	−1.2	0.81	−1.1	0.6	—	—	—	—	1	0.3	—	—
	Lobular (n = 21)	−1.1	0.7	1.2	0.2	1.1	0.4	—	—	—	—	−1	0.7	—	—

Histological types of 7 CC chemokines over-expressed in BrCa compared to normal tissues were determined utilizing all datasets available in ONCOMINE.

Bold represents significant RNA expression with a cut off of: fold change (FC) ≥ 1.4 and p-value ≤ 0.04 using t-test.

### CC Chemokine expression affects BrCa patient prognosis

**Figure 1:**
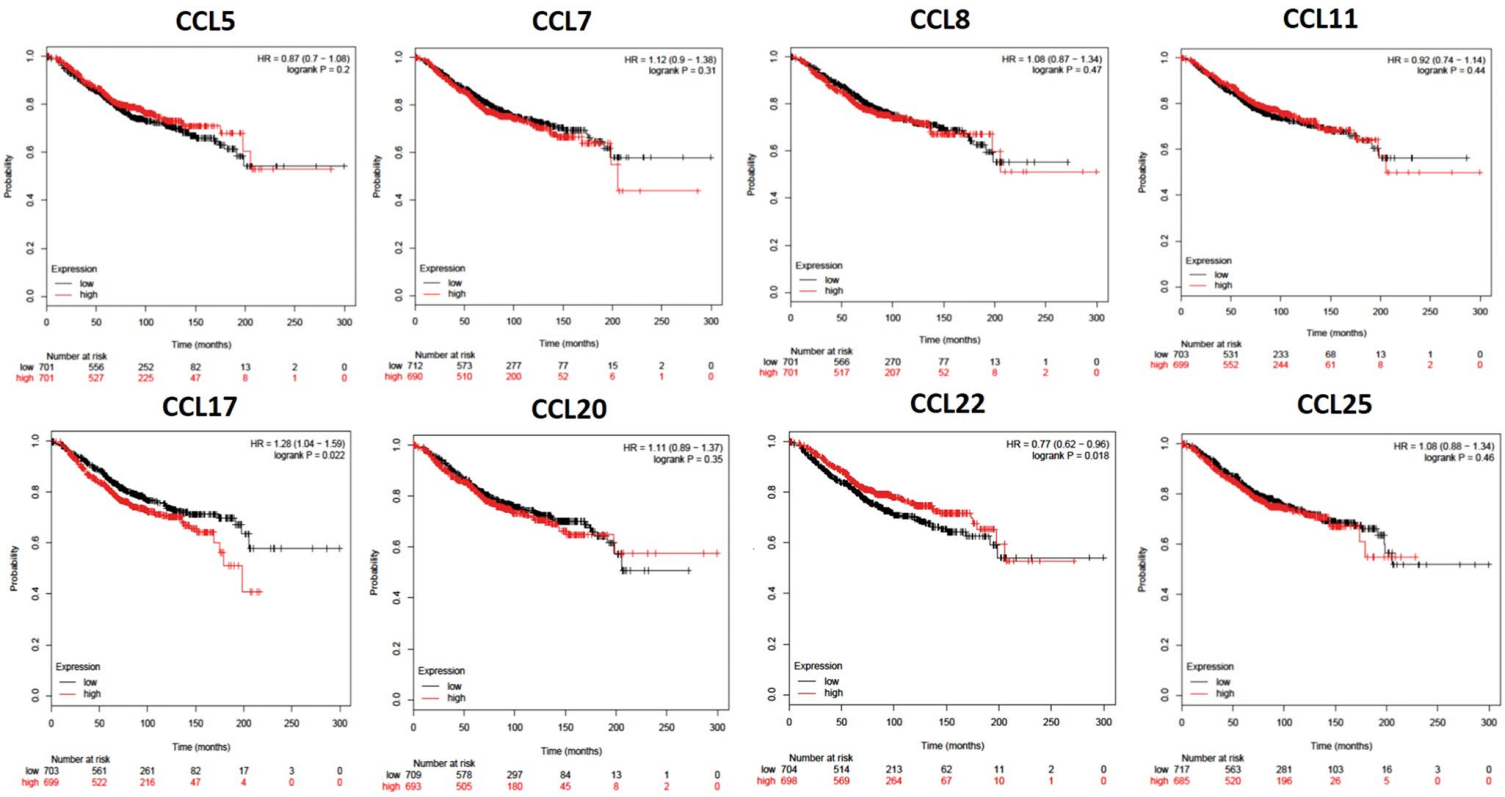
Kaplan-Meier curve of overall survival based on KMplotter mRNA gene chip expression of CC chemokines in BrCa patients. Red line represents higher expression and black line represents lower expression.

**Figure 2:**
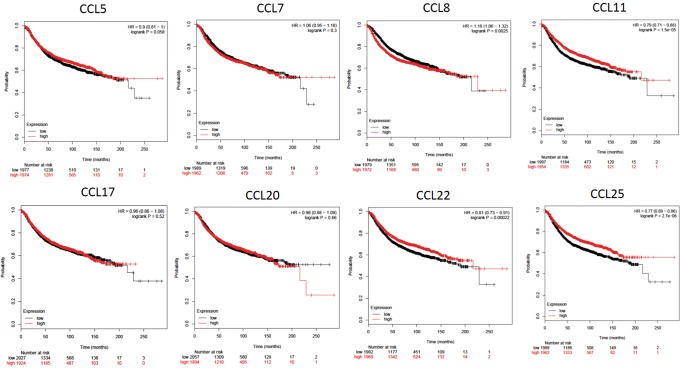
Kaplan-Meier Curve of relapse free survival based on KMPlotter mRNA gene chip expression of CC chemokines in BrCa patients. Red line represents higher expression and black line represents lower expression.

**Figure 3:**
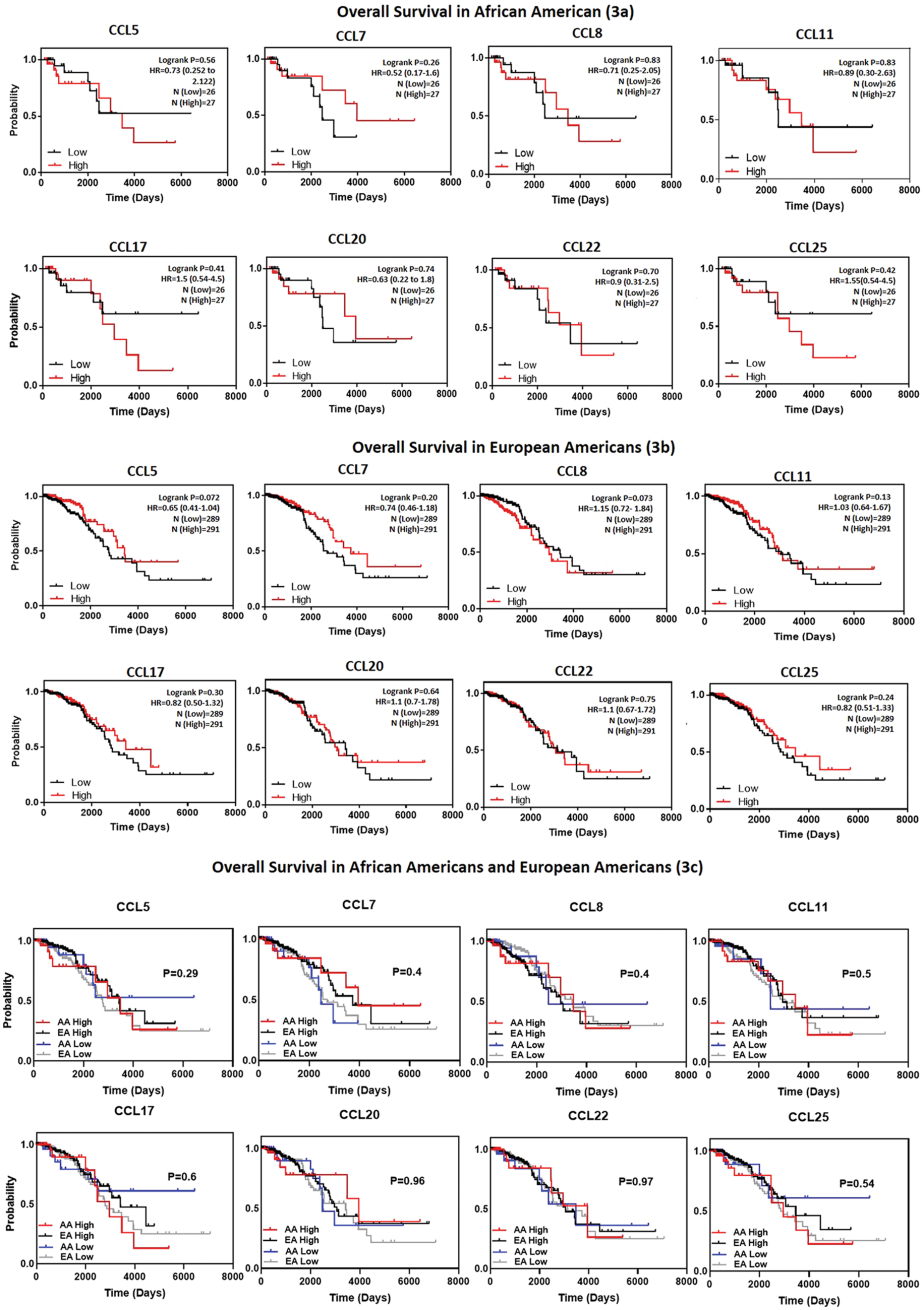
Kaplan-Meier curve of overall survival based on race using TCGA. In Panel a (AA) and Panel b (EA) red line represents higher expression and black line represents lower expression. Panel c shows AA vs. EA comparison. Red represents high expression in AA, black shows high expression in EA, blue represents low expression in AA and gray shows low expression in EA. Log-rank (Mantel-Cox) test was used to determine p-values and hazard ratio (HR).

To elucidate the prognostic significance of all CC chemokines in BrCa in regards to metastatic relapse the Bc-GenExMiner-v4.1 online database was used (Table 3). Significance (p < 0.04) was seen in CCL4 (p = 0.004, HR = 0.91), CCL5 (p = 0.034, HR = 0.94), CCL8 (p = 0.0017, HR = 1.1), CCL19 (p = 0.001, HR = 0.9), CCL21 (p = < 0.0001, HR = 0.9), CCL22 (p = < 0.0001, HR = 0.9) and CCL23 (p = 0.0014, HR = 0.9). CCL8 was the only one significantly associated with an increase in metastatic relapse (HR > 1.0). Next, we determined the association of CC chemokines, which were higher in BrCa (CCL5, -7, -11, -17, -20, -22 and -25) compared to normal tissues, with OS and relapse free survival (RFS) (Figs. 1 and 2) using KMplotter. We also included CCL8 due to its association with increased metastatic relapse. A trend towards a decrease in OS (higher expression, red line) was seen in CCL7 (p = 0.31, HR = 1.12), CCL8 (p = 0.47, HR = 1.06), CCL20 (p = 0.3, HR = 1.11), CCL25 (p = 0.45, HR = 1.08) and significantly in CCL17 (p = 0.022, HR = 1.28). On the other hand, CCL5 (p = 0.2, HR = 0.87), CCL11 (p = 0.44, HR = 0.82) and CCL22 (p = 0.016, HR = 0.77) were correlated with an increase in OS overall survival but the correlation was found to be significant only with CCL22. Decreased RFS was associated with CCL7 (p = 0.3, HR = 1.05). Interestingly CCL8 expression, which was not significantly elevated in BrCa tissues compared to normal tissues, showed an association with decreased RFS (p = 0.0025, HR = 1.18). Increased CCL11 (p = 1.5e^−05^, HR = 0.77), CCL22 (p = 0.0002, HR = 0.81) and CCL25 (p = 2.7e^−06^, HR = 0.77) expression were associated with an increase in RFS. We also determined the effect of CC chemokine expression on OS based on race (Fig. 3a (AA), 3b (EA) and 3c (AA vs. EA)). When CC chemokines were analyzed among these races, higher expression of CCL17 (p = 0.41, HR = 1.5) and CCL25 (p = 0.42, HR = 1.55) were associated with decreased OS in AA, whereas CCL8 overexpression was associated with decreased OS in EA (p = 0.073, HR = 1.15). To the contrary, high CCL7 (p = 0.26, HR = 0.52), CCL11 (p = 0.83, HR = 0.89) and CCL20 (p = 0.74, HR = 0.63) expression in AA were associated with increased OS. However, higher CCL5 (p = 0.072, HR = 0.65), CCL7 (p = 0.20, HR = 0.74) and CCL17 (p = 0.30, HR = 0.82) were associated with better prognosis (increase in OS) is EA women. It is important to note that CCL25 that is associated with poor prognosis (decreased OS) in AA (p = 0.42, HR = 1.55), was an indicator of better prognosis in EA (p = 0.24, HR = 0.82). When comparing association of CC chemokine expression among races (Fig. 3c), high expression of CCL17 (p = 0.6) and CCL25 (p = 0.54) in AA was associated with decreased OS however no such association of CCL17 and CCL25 expression was observed in EA. Lastly, high expression of CCL8 was associated with decreased OS in EA but not in AA (p = 0.4).

**Table 3:** Univariate Cox analysis of prognostic association of all 24 CC-Chemokine’s expression on metastatic relapse in BrCa based on the bc-GenExMiner v4.1 DNA gene chip database.

Chemokine	p value	HR	95% CI
CCL1	0.4894	0.98	0.92–1.04
CCL2	0.6384	1.01	0.96–1.08
CCL3	0.0785	0.91	0.83–1.01
**CCL4**	0.0038	0.91	0.86–0.97
**CCL5**	0.0335	0.94	0.88–0.99
CCL7	0.173	1.05	0.98–1.11
**CCL8**	0.0017	1.1	1.04–1.17
CCL11	0.4707	0.98	0.92–1.04
CCL13	0.9444	1	0.93–1.07
CCL14	0.5541	0.94	0.76–1.15
CCL15	0.1379	1.2	0.94–1.52
CCL16	0.6384	1.01	0.96–1.08
CCL17	0.8037	0.99	0.93–1.06
CCL18	0.6961	1.01	0.95–1.08
**CCL19**	0.001	0.9	0.85–0.96
CCL20	0.8989	1	0.94–1.07
**CCL21**	<0.0001	0.88	0.82–0.93
**CCL22**	<0.0001	0.87	0.82–0.93
**CCL23**	0.0014	0.88	0.81–0.95
CCL24	0.9997	1	0.94–1.07
CCL25	0.1064	1.05	0.99–1.12

HR-Hazard ratio. 95% CI- confidence interval. Bold represents a p value of ≤0.04.

### Association of CC chemokine expression with clinical parameters in BrCa patients

**Figure 4:**
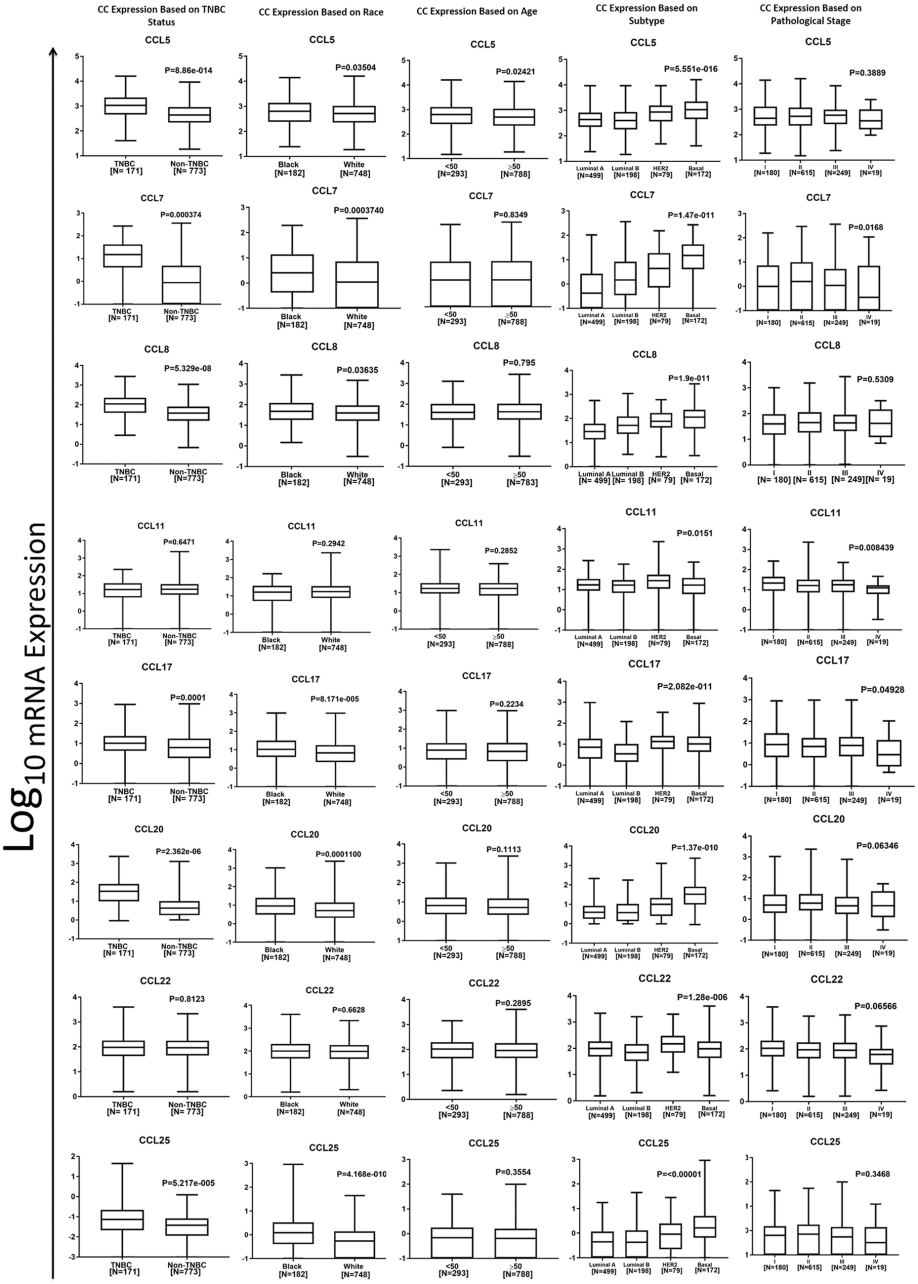
CC chemokines’ mRNA expression based on clinical parameters using TCGA. Box and whisker plots represent minimum expression at the bottom whisker, maximum at the top whisker and median at the middle line with a Log_10_ axis scale showing mRNA epxression of 8 CC chemokines. Statistical analysis was conducted with non-parametric Mann-Whitney for t-test and global significance for ANOVA with Kruskal Wallis.

Using TCGA data, we determined the association of CC chemokine expression with clinical parameters of BrCa patients (Fig. 4). Expression of CCL5 (p = 8.86e^−14^), CCL7 (p = 0.004), CCL8 (p = 5.33e^−08^), CCL17 (p = 0.001), CCL20 (p = 2.36e^−06^) and CCL25 (p = 5.22e^−05^) were highest in TNBC tissues compared to non-TNBC tissues. Additionally, expression of chemokines i.e. CCL5 (p = 5.55e^−016^), CCL7 (p = 1.47e^−011^), CCL8 (p = 1.9e^−11^), CCL20 (p = 1.37e^−010^), and CCL25 (p = < 0.00001) were highest in the basal BrCa subtype, when compared to luminal A/B and HER2 status. In HER2 positive BrCa tissues expression of CCL11 (p = 0.015) and CCL22 (p = 1.28e^−06^) were significantly higher. Out of the CC chemokines we have analyzed, CCL5 expression was the only one associated with age, where there was higher expression at a younger age (p = 0.024). Furthermore, CCL7 (p = 0.0004), CCL17 (p = 8.17e^−005^), CCL20 (p = 0.0001) and CCL25 (p = 4.2e^-01^), were significantly elevated in AA BrCa tissue compared to EA tissues. CCL8 (p = 0.04) and CCL5 (p = 0.04) expression in AA tissues were marginally higher in comparison to EA. Additionally, higher expression of CCL7 (p = 0.017), CCL8 (p = 0.53) and CCL25 (p = 0.35) showed a trend towards an increase in stage (from I to III). Such association was not seen with stage-IV likely due to very small sample size in this category.

## Discussion

Chemokines are predominantly known for their roles in immune cell trafficking and shaping the immune system. The role of chemokines and their corresponding receptors are well appreciated in cancer progression and metastasis^28^. Among all known chemokines, CXC chemokines are highly appreciated in cancer compared to CC chemokines. However, the CC chemokines are the largest family of chemokines and play an important role in inflammation^29^. Our laboratory was first to show association of CC chemokine receptor-9 in prostate, breast, ovarian and lung cancer^10,18–20^.

Studies have shown BrCa growth inhibition, after blocking CCR5 and CCR1, which are natural receptors for CCL5 and CCL7^30^. Chemokine receptors CCR1 and CCR5 are expressed on monocytes and facilitate their recruitment under chemotactic gradient of CCL5 and CCL7^31^ and play a crucial role in tumor progression. Our analysis showing higher CCL5 and CCL7 in BrCa tissues compared to normal tissues indicates that BrCa cells which produce high levels of CCL5 and CCL7 could be responsible for recruiting monocytes in the tumor microenvironment and supporting BrCa progression. In addition, production of CCL5 from mesenchymal stem cells (MSCs) results in BrCa production of colony-stimulating factor 1 (CSF1) under hypoxic conditions^32^. This causes the recruitment of myeloid-derived suppressor cells (MDSC) and tumor associated macrophages (TAM) to the tumor microenvironment^32^. Studies have shown that higher CCL5 promotes BrCa metastasis^33^. Our analysis also shows higher levels of CCL5 in medullary and ductal carcinoma and in TNBC compared to non-TNBC cases. Higher CCL5 may be involved in developing tumor tolerance resulting in the poor TNBC prognosis. As observed in our analysis, others have shown higher CCL5 in TNBC^34^. Non-remissive and later stage BrCa was reported to be correlated with CCL5 expression^35,36^, possibly due to its ability to promote pro-invasive factor MMP9 and monocyte migration to the BrCa tumor site, in which they undergo polarization allowing them to support tumor progression through angiogenesis^37^. This is not to our surprise as CCL5 was elevated in AA patients who often develop aggressive forms of BrCa. Furthermore, antagonizing CCR5 decreases CCL5 induced angiogenesis of BrCa cells^38^. CCL5 has been shown to promote BrCa progression in a p53 dependent manner through CCR5^39^. Interestingly, endothelial cells have been shown to increase metastasis of TNBC cells through secretion of PAI-1 and CCL5^40^. Hence our analysis showing higher CCL5 in BrCa (medullary and ductal carcinoma) compared to normal, higher CCL5 in TNBC compared to non-TNBC and higher CCL5 in AA compared to EA, suggests its potential contribution in shaping the tumor favoring microenvironment.

Our analysis also shows higher CCL7 in ductal breast carcinoma compared to normal tissues and other histological tissue types. It was also elevated in AA in comparison to EA patients. CCL7, that can bind and activate CCR1, CCR2 and CCR3, has been shown to promote metastasis by activating the MAPK cascade, promoting epithelial-mesenchymal transition (EMT) and CCR2+ TAM recruitment (enhancing vascular permeability^41^). Cancer associated fibroblast (CAF) derived CCL7 promotes BrCa proliferation^42^. Interestingly, CCL8 also known as monocyte chemo-attractant protein-2 (MCP-2), a natural ligand shared by CCR2, CCR3 and CCR5 was not elevated in BrCa tissues when compared to normal tissues, but was associated with poorer OS and RFS. It has been shown to drive BrCa metastasis. More specifically CCL8 stimulates fibroblasts generating a pro-tumor environment in the TNBC stroma, which was not seen in non-TNBC^43^. This was not to our surprise, as we found significantly higher CCL8 in TNBC tissues compared to non-TNBC tissues and it was associated with increased metastatic relapse. More importantly, when compared between AA and EA women, CCL8 was found higher in AA patients, who are frequently diagnosed with TNBC compared to EA. In contrast to its classical contribution on biology and outcome of BrCa, CCL8 was associated with poor OS in EA, suggesting race specific differences in CCL8 biology and immunity against BrCa.

Using tissue data we found that CCL11 (Eotaxin), which has a high affinity for CCR3, was higher in ductal breast carcinoma tissues when compared to healthy controls and other histological tissue types. Furthermore, a supporting study found that CCL11 was higher in the serum of BrCa patients when compared to serum of healthy individuals^44^. Bone colonization of BrCa cells is promoted by CCL11^45^, this is important to note as nearly 70% of BrCa cases show bone metastasis^46^. Co-culture studies showed an increase in CCL11 secretion by fibroblasts that enhanced chemoresistance and metastasis of BrCa cells^47^. In an allergic inflammation study, CCR3 antagonism prohibited chemotaxis of basophils and eosinophils^48^. Eosinophil degranulation has been reported in BrCa^49^, however this anti-tumor response is not quite understood. In pancreatic cancer basophil recruitment into tumor draining lymph nodes correlates with inflammation and poor survival^50^. Moreover, in hypoxic conditions tumor cells have been shown to secrete CCL11, recruiting CD206 expressing macrophages to the tumor, which in turn polarizes the classical macrophages to a M2 pro-angiogenic phenotype^51^. Clearly these facts and our data emphasize the need to study CCR3-CCL11 axis with respect to BrCa.

Furthermore CCR4 (receptor for CCL17 and CCL22) antagonists have been shown to decrease the tumor-promoting environment^52^. CCL17 recruits CCR4 positive regulatory T cells (Tregs) and promotes lung metastasis of BrCa by elimination of NK cells^53,54^. This coincided with our findings that higher CCL17 is expressed in BrCa and associated with decreased OS. Our data also shows CCL17 is overexpressed and is associated with poor prognosis in AA patients unlike EA. Its expression is higher in TNBC patients compared to non-TNBC patients. Moreover, CCR4 positively correlates with HER2 expression is BrCa cells^54^, supporting our result of higher expression of CCL22 in HER2 expressing tissues, when compared to basal and luminal types. We also found CCL22 to be significantly higher in BrCa (lobular) tissues compared to normal tissues, which agrees with a study reporting circulating CCL22 levels to be significantly higher in BrCa patients when compared to healthy controls^55^. As a prognostic factor in BrCa, tumor derived CCL22 has also been shown to activate and recruit CCR4 expressing Tregs^56,57^. Moreover, in prostate cancer, TAM promote tumor cell migration by activating CCL22-CCR4 signaling^58^. Additionally this axis has been shown to promote bone metastasis of lung cancer^59^. In contrast, our results showed that high CCL22 expression was associated with increased OS and RFS in BrCa.

The ligand for CCR6, CCL20 was higher in ductal and medullary breast carcinoma when compared to normal controls and other histological tissue types. Moreover, BrCa cells secrete CCL20, which recruits CCR6 expressing immune cells in the tumor vicinity^60^. Tumor cell produced CCL20/MIP3α allows them to attract CCR6 positive dendritic cell precursors^61^. Furthermore, CCL20 promotes migration and proliferation of surrounding breast cells^62^ and promotes BrCa initiation^63^. This could explain our finding showing higher expression of CCL20 in earlier stage I and stage II. Through paracrine signaling CCL20 promotes EMT in BrCa cells^64^. Studies have shown involvement of CCL20 in bone^65^ and lung^8^ metastasis of BrCa. We saw that higher expression of CCL20 associates with decreased OS. Moreover, expression of CCL20 was higher in AA with BrCa when compared to EA and in TNBC when compared to non-TNBC status. Chemotherapy has been shown to trigger higher production of CCL20 which in turn contributes to chemoresistance through ABCB1 drug efflux pump^66^, further suggesting involvement of CCL20 in disparities in therapeutic outcome.

CCR9 is selectively expressed on T cells and it’s only natural ligand (CCL25) is expressed in the thymus and small intestines^67^. CCR9 is often expressed on immature double (CD4+ and CD8+) positive thymocytes^68,69^, but has been reported to be expressed on thymocytes of all stages^70^. Circulating CCR9/CD4+ T cells exhibit a Th1 cytokine profile^71^. We found that CCL25 expression was associated with increased RFS when looking at all BrCa patients. We previously reported that CCL25 promotes migration/invasion^20^ and chemo-resistance^21^ in BrCa. Another study confirmed its role in BrCa and liver cancer migration and invasion^72^. Our current analysis further confirms our previous studies showing higher expression of CCL25 in BrCa compared to normal controls. Additionally, our data shows higher levels of CCL25 in TNBC compared to non-TNBC status, AA women compared to EA women and that it is associated with decreased OS in AA. Additionally, there was a trend of increased expression of CCL25 from stage I to stage III. Hence our current analysis explains a potential role of CCR9-CCL25 in disparities associated with poor overall outcome of BrCa in AA women.

It has been shown that chemokine receptors alone do not play a role in disease free or OS in BrCa^8^ (which we confirmed using Kmplotter, data not shown), suggesting the importance of their ligands. To our knowledge, this manuscript is the first to consolidate patient tissue data on CC chemokine ligands in BrCa and show that CC chemokine ligands are associated with disease free and OS. This suggests that chemokines expressed in the BrCa tissues not only support cancer cells expressing their corresponding receptors, but also shapes tumor microenvironment to overcome immune attack. However, these results should be confirmed and used with caution due to the fact that there are many factors downstream that could alter the effects of the variations seen at the mRNA level. In conclusion, identifying the role of chemokines in BrCa patients showing prominent expression could provide patient or race specific immunotherapeutic targets.

## Materials and Methods

### Oncomine

ONCOMINE^73^, online database (www.oncomine.org) of RNA/DNA sequencing data was used to screen all 24- CC chemokines’ RNA expression in BrCa tissues in comparison to normal tissues using the TCGA dataset (Table 1). Significant overexpression in BrCa tissues compared to normal tissues was determined using a t-test, utilizing a cut off of: fold change ≥1.4 and p-value ≤ 0.04. Additionally, these cut offs were used to classify significance for all downstream analysis. Further elucidation of histological  type of chemokines significantly expressed in BrCa (compared to normal tissues) was conducted with ONCOMINE utilizing all available datasets (Table 2). Using the referenced cutoffs, t-test were conducted comparing BrCa histological tissue types within each dataset.

### Bc-GenExMiner-v4.1

Microarray DNA expression results from BrCa Gene-Expression Miner (bc-GenExMiner)^74^ were used to classify prognostic association of metastatic relapse in BrCa of all 24 CC chemokines using univariate cox analysis (Table 3).

### Kaplan Meier Plotter-Breast Cancer

Association of CC chemokines on overall (Fig. 1) and relapse free (Fig. 2) survival was determined using Kaplan Meier (KM) Plotter (http://kmplot.com/analysis/)^75,76^. The two groups for plotting were high (red line) or low (black line) based on median mRNA gene chip expression.

### cBioPortal and Analysis

The cBio Cancer Genomics Portal (http://cbioportal.org)^77^ open-access database was used to download The Cancer Genome Atlas (TCGA) Pan Cancer Atlas dataset (RNASeq V2 RSEM) of 1084 BrCa patients (normalization and batch correction description here: https://www.synapse.org/#!Synapse:syn4976363). Graphpad Prism was used to produce Kaplan-Meir OS curves based on race (AA (Fig. 3a) or EA (Fig. 3b)), whereas red represents high expression and black low expression based on expression median. For AA vs. EA (Fig. 3c) survival curves red represents high expression in AA, black is high expression in EA, blue is low expression in AA and gray is low expression in EA. Log-rank (Mantel-Cox) test was used to determine p-values and hazard ratio. Expression based on clinical parameters was graphed as Log_10_ axis scale using box and whisker plots with whiskers at the minimum and maximum values and the middle line as the median (Fig. 4). Statistical analysis was conducted with non-parametric Mann-Whitney for t-test and global significance for ANOVA with Kruskal Wallis (Graphpad Prism7, La Jolla, CA). All statistics were thoroughly confirmed and verified by a statistician using SAS.

## Data Availability

All data generated or analyzed during this study are included in this published article.
